# How is COVID-19 affecting patients with obsessive–compulsive disorder? A longitudinal study on the initial phase of the pandemic in a Spanish cohort

**DOI:** 10.1192/j.eurpsy.2021.2214

**Published:** 2021-06-08

**Authors:** P. Alonso, S. Bertolín, J. Segalàs, M. Tubío-Fungueiriño, E. Real, L. Mar-Barrutia, M. Fernández-Prieto, S. Carvalho, A. Carracedo, JM. Menchón

**Affiliations:** 1 OCD Clinical and Research Unit, Psychiatry Department, Hospital Universitari de Bellvitge, Barcelona, Spain; 2 Institut d’Investigació Biomèdica de Bellvitge (IDIBELL), L’Hospitalet de Llobregat, Barcelona, Spain; 3 Department of Clinical Sciences, Bellvitge Campus, University of Barcelona, Barcelona, Spain; 4 CIBERSAM (Centro de Investigación en Red de Salud Mental), Instituto de Salud Carlos III, Madrid, Spain; 5 Genomics and Bioinformatics Group, Centre for Research in Molecular Medicine and Chronic Diseases (CiMUS), Universidade de Santiago de Compostela (USC), Santiago de Compostela, Spain; 6 Grupo de Medicina Xenómica, U‑711, Centro de Investigación en Red de Enfermedades Raras (CIBERER), Universidade de Santiago de Compostela (USC), Santiago de Compostela, Spain; 7 Fundación Pública Galega de Medicina Xenómica, Servicio Galego de Saúde (SERGAS), Santiago de Compostela, Spain; 8 Grupo de Genética, Instituto de Investigación Sanitaria de Santiago (IDIS), Santiago de Compostela, Spain; 9 Psychological Neuroscience Laboratory, CIPsi, School of Psychology, University of Minho, Campus de Gualtar, Braga, Portugal; 10 Department of Education and Psychology, University of Aveiro, Portugal; Department of Biology and William James Center for Research, University of Aveiro, Portugal

**Keywords:** COVID-19, obsessive–compulsive disorder, risk factors, social support, suicide

## Abstract

**Background:**

Although the consequences of the COVID-19 pandemic on emotional health are evident, little is known about its impact on patients with obsessive-compulsive disorder (OCD).

**Methods:**

One hundred and twenty-seven patients with OCD who attended a specialist OCD Clinic in Barcelona, Spain, were assessed by phone from April 27 to May 25, 2020, during the early phase of the pandemic, using the Yale–Brown Obsessive–Compulsive Scale (Y-BOCS) and a structured interview that collected clinical and sociodemographic information. Results were compared with those for 237 healthy controls from the same geographic area who completed an online survey.

**Results:**

Although 65.3% of the patients with OCD described a worsening of their symptoms, only 31.4% had Y-BOCS scores that increased >25%. The risk of getting infected by SARS-CoV2 was reported as a new obsession by 44.8%, but this only became the main obsessive concern in approximately 10% of the patients. Suicide-related thoughts were more frequent among the OCD cohort than among healthy controls. The presence of prepandemic depression, higher Y-BOCS scores, contamination/washing symptoms, and lower perceived social support all predicted a significantly increased risk of OCD worsening.

**Conclusions:**

Most patients with OCD appear to be capable of coping with the emotional stress of the COVID-19 outbreak and its consequences during the initial phase of the pandemic. Nevertheless, the current crisis constitutes a risk factor for a significant worsening of symptoms and suicidal ideation. Action is needed to ensure effective and individualized follow-up care for patients with OCD in the COVID-19 era.

## Introduction

On January 30, 2020, the World Health Organization declared the global COVID-19 outbreak to be an international public health emergency, and on March 11, declared that it had become a pandemic. As such, 2020 has seen social distancing and the adoption of strict hand and respiratory hygiene elevated as key strategies to control the spread of infection. There have been constant reminders for us all to wash our hands frequently and to avoid physical contact with other people and specific surfaces, behaviors that resemble those commonly exhibited by patients with obsessive–compulsive disorder (OCD) with contamination obsessions and washing compulsions.

Faced with the health, social, and economic consequences of the pandemic, almost 40% of the general population and more than 70% of health workers refer psychological distress, insomnia, anxiety, and depressive symptoms [1–4]. However, there is little data on how the current COVID-19 outbreak is affecting patients with OCD, a population that is more likely to have concerns about the risk of contracting contagious diseases [5, 6]. Two recent reports of patients with OCD from northern Italy describe a clinical worsening in more than a third of the subjects [7], and major vulnerability in those with contamination symptoms and in remission before the pandemic [8].

Beyond the fears related to the risk of contagion, potentially powerful stressors have arisen from the dramatic social and economic changes. These include home confinement, limited freedom, economic uncertainty, and difficulties accessing mental health services. For a stress-sensitive condition such as OCD, for which 60% of patients report stressful life events as a trigger for symptoms [9–11], the current milieu constitutes a period of serious vulnerability. At the same time, there are some important moderating features. Some patients may have had less need to face their fears thanks to the need for confinement during the pandemic. Moreover, there has been an acceptance and social normalization of some OCD behaviors, such as continuous hand washing or glove and sanitizer use, which were previously classified as pathological. Clearly, there are many unknowns about how patients with OCD have faced the pandemic and whether their disease protects against or worsens the negative psychological effects of the COVID-19 pandemic.

In this study, we aimed to evaluate the impact of the COVID-19 pandemic on a sample of patients with OCD at the initial stage of the health crisis, assessing not only changes in OCD severity, but also in pre-existing conditions, newly developed conditions, treatment, use of mental health resources, development of obsessive fears of SARS-CoV-2 contamination, and use of emotional regulation and stress coping strategies. Our hypothesis was that potential clinical and social predictive factors, both risk- and protection-related, including among others baseline OCD severity, specific symptom profiles, comorbid conditions, perceived social support or exposure to COVID-19 related news, could be established for changes in OCD status during the COVID-19 pandemic, allowing prevention strategies to be developed that focus on the most vulnerable patients.

## Method

### Participants and procedure

We invited adult outpatients who had been attending the specialist OCD Clinical and Research Unit of the Department of Psychiatry, Hospital de Bellvitge, Barcelona, Spain, for at least one year before March 2020 to participate in the study. Extensive clinical and sociodemographic information were available for these patients from before the pandemic. At an initial assessment, two independent psychiatrists with extensive clinical experience in OCD had checked that all participants fulfilled the diagnostic criteria for OCD according to the Structured Clinical Interview for The Diagnostic and Statistical Manual of Mental Disorders, Fifth Edition (DSM-5), Clinician Version [12]. After this first assessment, patients received pharmacological treatment and/or cognitive-behavioral therapy (CBT) according to international guidelines [13] and were asked to provide written informed consent to be included in a naturalistic follow-up cohort to study long-term prognosis of OCD. We excluded patients with psychoactive substance abuse/dependence (current or in the previous 6 months), psychotic disorders, intellectual disability, severe organic or neurological pathology (except tic disorders), or autism spectrum disorders at baseline from the cohort. The naturalistic cohort study involved quarterly visits in which obsessive and depressive symptoms were assessed using specific psychometric tools, with treatment adjusted according to the responses of each patient. For this study, we contacted those patients who had completed at least one year of treatment in our center before March 2020 and who had been on stable doses of medication for at least 3 months. This especially restrictive inclusion criterion was adopted to ensure that changes on OCD symptoms that could be detected at the initial stages of the pandemic were not affected by confounding factors such as recent changes on treatment.

Assessments were performed from April 27 to May 25, 2020. The requirement for strict confinement imposed by the Spanish government necessitated that we cancelled face-to-face hospital visits for psychiatric patients from March 15, 2020, in all but extreme urgency. As such, patients with OCD received outpatient follow-up by telephone. This included interviews and clinical assessments by their regular psychiatrist to determine the need for treatment adjustment. It was during these telephone consultations that eligible patients were given the option to participate in the study. After informing patients about the study’s purposes and characteristics, oral consent was obtained and recorded in their medical history. Those who agreed to participate then underwent a structured interview specifically designed for the study. This lasted 20–25 min and was also conducted by their regular psychiatrist.

Participants from the general population were recruited through social networks, using a snowball method. Requirements for inclusion in the controls group were age >18 years old, no history of psychological or psychopharmacological treatment, no prior diagnosis of a mental disorder, and living in Catalonia throughout the COVID-19 pandemic. An adapted version of the structured interview delivered to the OCD group (excluding OCD-related questions) was sent as an online survey via social networks to participants in the control group, who provided written informed consent to participate in the study. Snowball sampling has the drawback, like all nonrandom sampling, of not guaranteeing the representativeness of the population. To reduce this risk, an initial selection of individuals was designed to guarantee access to subjects of all cultural and socioeconomic levels of the population, requesting the participation of cleaning kitchen and maintenance personnel of the university and hospital, health workers of all levels and blood bank donors, and the contacts of all of them. The rate of a number of unemployed subjects (16%) coinciding with the unemployed rate in Spain at the initial stages of the pandemic, suggests that the group of healthy controls can be considered representative of the socioeconomic status of the general population.

The study was approved by the Ethics Committee of the Hospital de Bellvitge and was performed in accordance with the principles established by the declaration of Helsinki, as revised in 1989.

### Measures

The structured interview that we designed specifically for this study assessed seven thematic blocks:

1. Sociodemographic data: age, gender, civil status, educational level, employment situation, changes in employment, and/or income during the pandemic.
2. Social context: type of family coexistence (living alone, with birth family, own family, or friends), perceived family and social support (yes/no on whether you perceive you have (a) someone to confidently talk, (b) someone to provide company, and (c) environment offers help and support), leaving home pattern during the pandemic, time spent listening/reading to news about the pandemic and perceived changes on family atmosphere and harmony during the pandemic (no change/better/worse).
3. Contact with COVID-19: confirmed or suspected COVID-19, hospitalization related to COVID-19, and admission to intensive care unit due to COVID-19 in the subject/family/close friends; COVID-19 death of a family member or close friend.
4. Medical conditions: chronic medical conditions (including respiratory diseases, obesity, diabetes, hypertension, kidney or liver failure, immunodeficiencies, cancer, and/or transplants); use of tobacco, alcohol, and drugs before and during the pandemic, categorically (yes/no) and continuously assessed (number of daily tobacco or cannabis cigarettes, daily standard drink units or weekly episodes of cocaine, heroin, or psychoactive drugs use).
5. Emotional regulation and stress coping strategies: worries about getting COVID-19; worries about family members getting COVID-19; concerns about home isolation and loss of routines, difficulties contacting mental health services (for the patients with OCD), separation from loved ones and economic consequences of the pandemic were assessed through 0–4 Likert scales with 0 = no or very little, 1 = little, 2 = some, 3 = quite, and 4 = a lot. Use of stress coping strategies included questions (yes/no) on physical exercise practice, mindfulness/meditation practice, yoga, relaxation techniques, using of online social/family networking, establishing a daily routine and hobbies, and distracting activities.
6. General psychiatric data: perceived changes in eating patterns (no change and increased/decreased appetite) and sleeping patterns (no change, early/middle/late insomnia, nightmares, and need medication to sleep), depression and anxiety levels since March 14, 2020, according to a visual analog scale (VAS; 0 = no anxiety/no depressive symptoms and 10 = severely anxious/depressed); suicidal ideation according to the Hamilton Depression Rating Scale (HDRS; item on suicide) [14]. Development of new psychiatric symptoms was assessed by a battery of questions adapted from the Structured Clinical Interview for DSM-5-Clinician version [12] including questions on depression, manic episodes, panic attacks, generalized anxiety, specific phobias, social phobia, psychotic symptoms, and impulse control disorders, such as compulsive buying, compulsive Internet use, and pathological gambling.
7. OCD-related questions (OCD group only): changes needed for the treatment of OCD during the pandemic, need for urgent care or psychiatric hospitalization during the pandemic, changes in OCD severity according to the clinician-administered Yale–Brown Obsessive–Compulsive Scale (Y-BOCS)[15], changes in OCD symptomatology according to the Y-BOCS symptom checklist (previous and newly developed symptoms during the pandemic with a specific question on fears of contamination by SARS-CoV2). The Y-BOCS rates obsessions and compulsions separately on five dimensions: time spent, degree of interference, distress, resistance and perceived control over symptoms. The 10 Y-BOCS items are each scored on a 4-point scale from 0 (none) to 4 (extreme). Total score ranges from 0 to 40 (0–7: subclinical; 8–15: mild; 16–23: moderate; 24–31: severe; and 33–40: extreme OCD).

Clinical data were available for patients in the OCD group just before the pandemic outbreak in March 2020. This included scores on the clinician-administered HDRS, Y-BOCS, and Y-BOCS symptom checklist (last assessment between December 14, 2019 and March 14, 2020); age at OCD onset (defined as the moment when obsessive symptoms reached a clinically significant level); and OCD treatment, including type, dose, and duration of pharmacological treatment and/or previous or ongoing CBT.

### Statistical analysis

Descriptive statistics were calculated and are reported. Independent sample Student *t*-tests and Pearson Chi-squared tests were carried out to explore the differences in sociodemographic and clinical variables between the OCD and control groups. Paired sample Student *t*-tests were also performed to assess potential changes in OCD severity (obsessions, compulsions, and total Y-BOCS scores) from before to after the COVID-19 outbreak.

To explore the potential predictors of changes in OCD during the pandemic, we first performed bivariate analysis to assess the association of each sociodemographic, pandemic-related, and clinical variable with changes on the Y-BOCS scores from before the pandemic to assessment in April or May 2020. In a second step, we conducted a generalized linear regression analysis with changes on Y-BOCS scores as the dependent variable and those variables significantly associated with these changes in the bivariate analysis as independent variables (prepandemic HDRS score, prepandemic Y-BOCS score, contamination or cleaning symptoms and perceived social support). Significance was set at *p* < 0.05 and all analyses were carried out using IBM SPSS Version 24 for Windows.

## Results

### Samples characteristics

We included 364 subjects in the study (127 OCD patients and 237 healthy controls). Six more OCD patients who fulfilled inclusion criteria could not be contacted and three patients declined to participate in the study because they preferred not to discuss their symptoms over the phone (response rate 93.3%). Since the healthy control group was recruited through the snowball method, we lack information about how many subjects received the interview and refused to complete it.

Before the pandemic, 33 (25.9%) patients with OCD fulfilled criteria for remission, showing residual obsessions and compulsions that did not interfere with their everyday lives (Y-BOCS scores <12) [16]. The sociodemographic characteristics of the two groups are described in Table 1. Significant differences between the OCD and control groups were detected for educational level, working status, family structure, changes in economic income, perceived support, and effect on family life during the pandemic. Patients with OCD had completed fewer years of education (12.7 ± 2.8 vs. 14.6 ± 2.6) and developed paid work activity less frequently than healthy subjects, with 54% of them being unemployed or receiving a disability pension. These results are consistent with those from other cohorts of OCD patients and with recent studies indicating that OCD is associated with lower educational attainment and marked difficulties to participate in the labor market [17, 18]. Healthy controls reported more perceived emotional support, more changes in their economic income, and less worsening of family life during confinement than OCD patients.

**Table 1.** Social and clinical characteristics of OCD patients and healthy controls.

Variables		OCD (*n* = 127)	Control (*n* = 236)	*t* or *Χ*^2^	*p*	95% Interval confidence
						Lower/upper
Age, years (mean ± SD)		42.0 ± 11.3	40.8 ± 12.3	0.9^a^	0.3	−1.32 3.86
Years of education, years (mean ± SD)		12.7 ± 2.8	14.6 ± 2.6	−6.3^a^	<0.001	−2.4 −1.31
Age at OCD onset, years (mean ± SD)		17.5 ± 6.2				
Gender (male/female)		59/68	100/136	0.5	0.4	
Civil status, *n*, %	Single	55 (43.3%)	83 (35.1%)	5.5	0.1	
	Married	59 (46.4%)	133 (56.3%			
	Divorced/widowed	13 (10.2%)	20 (8.4%)			
Working status, *n*, %	Paid employment	44 (34.6%)	69 (29.2%)	73.9	<0.001	
	Self-employment	10 (7.8%)	82 (34.7%)			
	Students	4 (3.1%)	0 (0%)			
	Unemployed	16 (12.5%)	38 (16.1%)			
	Pensioners	53 (41.7%)	1 (0.4%)			
Living status, *n*, %	Alone	17 (13.3%)	17 (7.2%)	10.3	0.01	
	Birth family	40 (31.4%)	50 (21.1%)			
	Own family	67 (52.7%)	161(68.2%)			
	With friends	3 (2.3%)	8 (3.3%)			
Living with other mentally ill subjects, yes, *n*, %		14 (11.0%)	13 (5.5%)	3.6	0.05	
Living with dependents (children, parents), yes, *n*, %		45 (35.4%)	75 (31.7%)	0.4	0.4	
Perceived support sources, yes, *n*, %	Someone to confidently talk	103 (81.1%)	226(95.7%)	20.9	<0.001	
	Someone to provide company	102 (80.3%)	221(93.6%)	14.9	<0.001	
	Environment offers help and support	109 (85.8%)	210(88.9%)	0.7	0.3	
Income, *n*, %	No change	100 (78.7%)	136(57.6%)	25.0	<0.001	
	20% reduction	0 (0%)	30 (12.7%)			
	20–50% reduction	14 (11.0%)	29 (12.2%)			
	>50% reduction	10 (7.8%)	28 (11.8%)			
	Increase	3 (2.3%)	13 (5.5%)			
Exists during confinement, *n*, %	Never	32 (25.1%)	0 (0%)	201.9	<0.001	
	1–2/week	62 (48.8%)	13 (5.5%)			
	3–6/week	4 (3.1%)	103(43.6%)			
	Daily	33 (25.9%)	120(50.8%)			
Family harmony *n*, %	No change	60 (47.2%)	113(47.8%)	10.0	0.007	
	Better	32 (25.1%)	88 (37.2%)			
	Worse	35 (27.5%)	36 (15.2%)			

Abbreviation: OCD, OCD, obsessive–compulsive disorder.

a Results by *t*-test; all others are by χ^2^.

### Direct contact with SARS-CoV2 infection

No significant differences were detected between the OCD and control groups in personal or family history of confirmed or suspected SARS-CoV2 infection, as well as hospital and intensive care unit admissions. The same was true for the number of family members or friends who died from COVID-19. However, healthy controls more frequently reported having close friends diagnosed with COVID-19 and/or needed to be admitted to hospital or intensive care units (Supplementary Table S1).

### Psychopathology during the pandemic

Psychopathology arose from different domains in each group during the pandemic, as described in Table 2. Compared with controls, the OCD group scored higher on perceived levels of anxiety and depression, had more frequent suicide-related thoughts, and more often experienced changes in perceived eating and sleeping patterns. The appearance of depressive symptoms that subjectively affected the individual’s functionality was more frequent in the OCD group, while the control group described more pathological behaviors related to impulse control, including pathological gambling, compulsive internet use, or compulsive buying. Healthy subjects also consumed more alcohol before the pandemic than the OCD group and tended to increase consumption significantly during the confinement. No significant differences were detected for tobacco or other drugs use.

**Table 2.** Psychopatology during the pandemic among OCD patients and healthy controls.

Variables		OCD (*n* = 127)	Controls (*n* = 237)	*t* or *Χ*^2^	*p*	95% Interval confidence
						Lower/upper
Anxiety (VAS)		6.0 ± 2.3	3.3 ± 2.6	4.9	<0.001	2.0 3.1
Depression (VAS)		5.4 ± 2.5	2.2 ± 2.3	0.9	<0.001	2.7 3.7
Fear of getting COVID-19		2.8 ± 1.3	2.2 ± 0.8	5.1	<0.001	0.37 0.83
Fear of family members getting COVID-19		3.7 ± 1.1	3.1 ± 1.0	5.1	<0.001	0.38 0.85
Worried about confinement		2.8 ± 1.1	2.3 ± 1.0	4.3	<0.001	0.27 0.74
Impossibility of family contact		3.1 ± 1.1	2.9 ± 1.0	2.1	0.03	0.01 0.48
Economic consequences		2.9 ± 1.1	2.8 ± 1.1	1.1	0.2	−0.11 0.39
Daily hours listening/reading COVID-19 related news		1.6 ± 1.6	0.8 ± 1.0	6.2	<0.001	0.6 1.1
Drug consumption, *n*, %	Tobacco	28(22.0%)	56(23.7%)	0.1	0.7	
	Alcohol	5 (3.9%)	77(32.6%)	38.6	<0.001	
Other drugs	Cannabis	2 (1.5%)	9 (3.8%)	3.4	0.3	
	Cocaine	1 (0.7%)	1 (0.4%)			
	Other drugs	0 (0%)	0 (0%)			
Increase in drug consumption, *n*, %	Tobacco	15(11.8%)	18 (7.6%)	1.7	0.1	
	Alcohol	5 (3.9%)	23 (9.7%)	3.8	0.04	
	Other drugs	0 (0%)	3 (1.2%)	1.6	0.2	
Suicide, *n*, %	Absent	90(70.8%)	231 (97.8%)	56.1	<0.001	
	Feels life is not worth living	25(19.6%)	4 (1.6%)			
	Wishes he/she were dead	5 (3.9%)	1 (0.4%)			
	Ideas or gestures of suicide	7 (5.5%)	1 (0.4%)			
	Attempts at suicide	0 (0%)	0 (0%)			
Eating pattern, *n* %	No change	48(37.7%)	101 (42.7%)	13.0	0.04	
	Increase appetite	50(39.3%)	101 (42.7%)			
	Decreased appetite	29(22.8%)	35(14.8%)			
Sleep pattern, *n*, %	No change	59(46.4%)	110 (46.6%)	13.2	0.02	
	Early insomnia	33(25.9%)	63(26.6%)			
	Middle insomnia	24(18.8%)	46(19.4%)			
	Early wake-up	3 (2.3%)	16 (6.7%)			
	Nightmares	3 (2.3%)	2 (0.8%)			
	Need medication to sleep	4 (3.1%)	0 (0%)			
Coping strategies,^a^ *n*, %	Physical exercise	61(48.0%)	179 (75.8%)	27.8	<0.001	
	Meditation	23(18.1%)	53(22.4%)	0.9	0.3	
	Yoga	12 (9.4%)	59 (25%)	12.5	<0.001	
	Muscular relaxation	32(25.1%)	53(22.4%)	0.3	0.4	
	Distracting activities	62(48.8%)	115 (48.7%)	0.003	0.9	
	Time routine	74(58.2%)	164 (69.4%)	4.3	0.03	
	Online or real conversations	105 (82.6%)	199 (84.3%)	0.1	0.7	
New psychiatric difficulties during confinement, *n*, %	Depression	41(32.2%)	9 (3.8%)	61.2	<0.001	
	Panic attacks	1 (0.7%)	4 (1.6%)			
	Excessive worrying (GAD)	5 (3.9%)	11 (4.6%)			
	Specific phobias	0 (0%)	1 (0.4%)			
	Eating disorders	0 (0%)	2 (0.8%)			
	Pathological gambling	0 (0%)	2 (0.8%)			
	Compulsive buying or web use	4 (3.1%)	7 (2.9%)			
	Compulsive pornography use	1 (0.7%)	2 (0.8%)			

Abbreviations: GAD, generalized anxiety disorder; OCD, obsessive-compulsive disorder; VAS visual analog scale.

a Results not adjusted for other variables.

### Emotional regulation and stress coping strategies

Patients with OCD were more concerned than healthy controls about the risks to themselves or their families of being infected by SARS-CoV2 and the loss of their daily routines due to the pandemic. Both groups had similar concerns about the economic consequences of the crisis and loss of family contact. Regarding coping strategies, patients with OCD tended to engage in less physical exercise and yoga than healthy controls and reported that it was more difficult for them to establish a daily routine. Both groups used online or face-to-face conversations as the main coping strategies for coping with emotional distress (Table 2).

### Changes on OCD symptomatology

A statistically significant increase in Y-BOCS scores was detected in the OCD sample during the first months of the pandemic (*t* = −8.3, *p* < 0.001), with an average 2.7-point increase in Y-BOCS scores. It represents a 15.1% increase from baseline scores, a percentage that can be considered of little clinical relevance (clinical changes of at least 25% are required for example as an operational criteria for partial response [16]). No significant differences were detected when comparing patients who met the pre-pandemic criteria for remission and those who experienced time-consuming and life-interfering symptoms before the COVID-19 outbreak (*t* = −0.4, *p* = 0.6).

Although 83 patients (65.3%) described a worsening of their symptoms, only 40 (31.4%) showed an increase of >25% from their pre-pandemic Y-BOCS scores, indicating at least moderate clinical relevant repercussion (Table 3). In this group, an average increase on Y-BOCS scores of 7.0 points ± 2.21 (range: 4–12) was detected, with Y-BOCS scores increasing from mean scores of 18.1 ± 4.1 at prepandemic assessment to 25.1 ± 5.4 after the COVID outbreak, which indicates moving from suffering a moderate OCD to a severe form of the disorder. It was notable that 20 patients (15.7%) described a significant improvement in their obsessions and compulsions, experiencing a mean decrease in their Y-BOCS scores of 11.1%. Although 57 patients (44.8%) reported new obsessions and compulsions specifically related to the risk of contamination by SARS-CoV-2, this only became the main obsessive concern for 12 (9.4%). Obsessive fears about COVID-19 occurred more frequently among those who reported contamination/washing symptoms before the pandemic than those with other symptom dimensions. Indeed, 78.9% reporting this fear as a new symptom already suffered from contamination obsessions and compulsions (increasing to 83.3% for those in whom this fear became the main obsession). Pharmacological treatment needed to be changed in the first months of the pandemic for 32 (25.1%) patients, with all but two of these receiving increased selective serotonin reuptake inhibitor or clomipramine doses (*n* = 16), increased antipsychotic doses (*n* = 3), or the addition or increase of benzodiazepine doses.

**Table 3.** Changes in OCD symptomatology and related conditions.

HDRS baseline (m, SD, range)		10.8 ± 5.3 (2–22)
Y-BOCS baseline	Obsessions	9.0 ± 3.1 (3–17)
	Compulsions	8.8 ± 3.1 (2–17)
	Total	17.8 ± 6.2 (6–34)
Y-BOCS confinement	Obsessions	10.4 ± 3.7 (3–17)
	Compulsions	10.0 ± 3.9 (2–17)
	Total	20.5 ± 7.5 (6–34)
Self-perception of OCD worsening (VAS)		2.9 ± 3.0
Change on Y-BOCS score (*n*,%)	Improvement (*n* = 20, 15.7%)	11.1 ± 5.0 (3.5–16.4)
mean ± SD (range)	No change (*n* = 24, 18.8 %)	
	Worsening (*n* = 83, 65.3%)	25.6 ± 16.1 (4.5–87.5)
Worsening >25% Y-BOCS	Yes (*n*, %)	40 (31.4%)
Contamination/cleaning baseline (n, %)	Absent	52 (40.9%)
	Present	32 (25.1%)
	Principal	43 (33.8%)
Aggressive/checking baseline	Absent	35 (27.5%)
	Present	35 (27.5%)
	Principal	57 (44.8%)
Symmetry/ordering baseline	Absent	79 (62.2%)
	Present	28 (22.0%)
	Principal	20 (15.7%)
Sexual/religious baseline	Absent	106 (83.4%)
	Present	9 (7.0%)
	Principal	12 (9.4%)
Obsessive fears of COVID-19 contamination	Absent	70 (55.1%)
	Present	45 (35.4%)
	Principal	12 (9.4%)
Medication status baseline	SSRIs	53 (41.7%)
	Clomipramine	18 (14.1%)
	SSRI or clomipramine + antipsychotic	56 (44.0%)
Previous or current CBT baseline		105 (82.6%)
Medication change during confinement	Increase SSRI or clomipramine	16 (12.5%)
	Decrease SSRI or clomipramine	2 (1.5%)
	Increase antipsychotic	3 (2.4%)
	Add or increase benzodiazepine	11 (8.6%)

Abbreviations: CBT, cognitive-behavioral therapy; HDRS, Hamilton Depression Rating Scale; OCD, obsessive-compulsive disorder; SSRI, selective serotonin reuptake inhibitors; VAS visual analog scale; Y-BOCS, Yale–Brown Obsessive–Compulsive Scale.

Linear regression analysis showed that the following significantly predicted a worsening of OCD symptomatology during the first months of the COVID-19 outbreak (*R*^2^ = 0.30; *F* = 10.2): prepandemic HDRS (*p* < 0.001) and Y-BOCS scores (*p* = 0.006); the presence of contamination/washing obsessions and compulsions (*p* = 0.002); and lower perceived social support (*p* = 0.04) (Table 4).

**Table 4.** Predictors of changes on OCD symptoms during the pandemic.

Predictors	Non-standardized coefficient	Standardized coefficient	Wald’s 95% confidence interval for *B*		t	*p*-value
	*B*	beta	Lower	Upper		
HDRS	1.7	0.47	1.2	2.3	6.22	<0.001
Contamination/cleaning pre-pandemic symptoms	9.5	0.23	3.4	15.6	3.11	0.002
Y-BOCS pre-pandemic	0.69	0.21	0.20	1.19	2.81	0.006
Social support	−3.2	−0.15	−6.4	−0.11	−2.05	0.04

Abbreviations: HDRS, Hamilton Depression Rating Scale; OCD, obsessive–compulsive disorder; Y-BOCS, Yale–Brown Obsessive–Compulsive Scale.

## Discussion

In the present study, patients with OCD experienced a moderate worsening of their symptomatology during the first months of the pandemic. Increases in obsessions and compulsions were especially relevant for those with more severe forms of OCD, associated depressive symptoms, contamination or washing obsessions and rituals, and less perceived emotional support before the pandemic. It was notable that patients with OCD were at increased risk of suicidal thoughts and/or acts and changes on perceived eating and sleeping patterns, and that they had higher perceived levels of anxiety and depression, compared to healthy controls. These last ones, on their hand, exhibited more pathological behaviors related to impulse control including pathological gambling, compulsive internet use, or compulsive buying. These results coincide with the risk indicated by the experts, on some vulnerable subjects, of developing addictive disorders as a consequence of the inappropriate use of online activities such as gambling, shopping, video gaming or social media use, as dysfunctional strategies to reduce emotional distress or improve mood [19].

The percentage of patients with OCD in our sample who showed an increase in symptoms (65.3%) was similar to that described after online surveys by Jelinek et al. [20] in a sample of 394 OCD patients from Germany (72%) and by Wheaton et al. in a group of 252 self-identified adults with OCD from the United States (76.2%) [21]. In all three cases, this percentage was significantly higher than the 35.8% reported by Benatti et al. [7] in northern Italy. However, the Italian study lacked a psychometric assessment and reported only on “global clinical worsening,” a concept that might better corresponds to the 31.4% of our patients who presented a worsening of Y-BOCS scores >25%, a value associated with a significant functional impairment. It is interesting that the net Y-BOCS increases between our study and that of Davide et al. [8] are comparable, implying similar outcomes for OCD in countries that have similar experiences of the pandemic.

It is also remarkable that 15% of our patients described a significant improvement in symptoms during the pandemic and that another 20% reported that they remained stable. These results are in the same line as those described in a recent study of three Dutch psychiatric cohorts, including a sample of 130 OCD patients, which describes that patients with more severe and chronic forms of mental illness showed a reduction in the severity of their symptoms during the first weeks of the pandemic [22]. Although we cannot exclude that home isolation led to some indirect avoidance of obsession-inducing situations, our results suggest that many patients with OCD could adapt to the great personal and social changes forced on them by the pandemic. In fact, resilience in the face of potential trauma may be more common than is often believe [23]. This ability to cope with difficulties is mediated by multiples determinants, with perceived social support being among the most important protective factors against posttraumatic stress both in general [24] and in front of the COVID-19 pandemic [25]. Patients with OCD in our sample perceived less emotional support than the healthy controls, and this variable was associated with a higher risk of symptom deterioration during the pandemic. This stresses the importance of promoting all methods of social support, including video calls with friends and family. Crucially, access to self-help groups and community resources was drastically reduced during the first months of the pandemic in Spain. Perhaps this decision should be reassessed to reduce feelings of loneliness and improve social support in psychiatric patients.

Among the patients with OCD, those with symptoms related to contamination/cleaning were most vulnerable to clinical deterioration. In this group, 60% reported developing new obsessions related to the fear of infection by the SARS-CoV-2 virus, with this becoming the main concern for 13.3%. By contrast, this concern was significantly less common for patients with other clinical presentations. This issue constitutes a therapeutic challenge in the context of the current pandemic because contamination obsessions and washing compulsions tend to respond especially well to exposure response prevention [26]. At present, CBT must be used with caution because of the potential for increased risk of contagion by the SARS-CoV-2 virus [27]. It is critical that we develop guidelines for exposure and response prevention therapy that allow clinicians to conduct exposure therapy responsibly, with special attention to local COVID-19 risk [28]. However, while the pandemic is not controlled, other therapeutic approaches may be more appropriate, including imaginal exposure, danger ideation reduction therapy, behavioral activation, and activity scheduling [27]. Activity scheduling, in particular, may be especially relevant given our finding that patients with OCD have more difficulties than the general population in maintaining a healthy and regular daily routine during quarantine and home isolation.

Although none of the patients in our sample attempted suicide during the first weeks of the pandemic, 5.5% described active suicidal thoughts and 23% had passive suicidal ideation. These percentages were clearly higher than those in the general population, highlighting a need for careful assessment of suicide risk in patients with OCD during the COVID-19 pandemic [27]. Given that face-to-face visits have been drastically reduced, it is essential to ensure close follow-up of OCD patients through telemedicine resources, something that it is not easy in elderly cohorts, or in those with either lower educational levels or difficulties in accessing technological resources [29]. Ensuring safe access to urgent psychiatric care is also essential for patients with OCD, not least because they may have fears of approaching hospitals due to the fear of contagion.

One in four patients in our study required medication adjustments during the first months of the pandemic. In the study by Benatti et al. [7] this was necessary for a 35.7% of their sample and increased to 70.5% in the subgroup that experienced a global worsening of OCD. These therapeutic needs must be taken into account despite the complex health situation that the pandemic entails. As with psychiatric services across the world, we have had to modify our practice to guarantee proper care and support to our OCD patients. Therapeutic gaps have been filled by improving the ease of telephone or email contact between therapists and patients, introducing periodic phone conferencing with patients, and delivering internet-based CBT. Nevertheless, we must consider that some patients may find it difficult to follow remote therapies, especially those of advanced age, with fewer economic resources, or with less technological skill, and may therefore disengage from treatment. More than ever, individually tailored treatments are crucial [30].

The current study presents several limitations. The OCD group attended a specialist OCD clinic in a tertiary hospital and may not reflect patients with OCD in the general population, either in terms of severity or access to specific treatments. Nevertheless, our sample included patients with both mild and severe forms of the disorder, with a quarter of the patients meeting the criteria for remission prior to the pandemic. This also applies to the control group who, in addition to the limitations derived from its obtaining by the snowball system, were chosen through social networks and completed an online interview, limiting access to those who use this technology. In this sense, OCD patients were interviewed by telephone while healthy subjects answered the same questions through an online questionnaire. Although the formulation of the questions was identical in both versions, it cannot be ruled out that this methodological difference might influence our results. Nevertheless, the clinical assessment of OCD patients by a specialist through telephone interview is one of the strengths of our study, at a time when most published results from clinical populations are obtained only by online interviews. Data were collected in a cross-sectional assessment and only cover the period to May 2020. It is possible that patients with OCD adapted better in the early phases of confinement when government control measures were quite clear and restrictive, but they may have more difficulties managing later phases of de-confinement when the margin for personal responsibility is much greater. Prospective data are being acquired in this same sample to resolve this concern and assess the long-term impact of COVID-19 in the OCD group. The development of obsessive fear of COVID-19 contamination in healthy subjects was not explored due to the difficulty in differentiating them from normal fear of contagion in an online interview. In this sense, whether the current pandemic will prove to be a turning point that increases the global risk of OCD is still unknown.

Our results suggest that most patients with OCD have been able to cope adequately with the emotional, social, and economic sequelae associated with the COVID-19 outbreak, at least in the early stages of the pandemic. However, the current crisis has constituted a risk for significant worsening, including the development of depressive and suicidal symptoms, for one in three patients. Actions must be taken to guarantee continuous follow-up and access to adequate and individualized treatment for patients with OCD while the COVID-19 pandemic is ongoing.

## Data Availability

The data that support the findings of this study are available from the corresponding author upon reasonable request.

## Supplementary material

For supplementary material accompanying this paper visit http://dx.doi.org/10.1192/j.eurpsy.2021.2214. click here to view supplementary material
