# Supplementary material for: How is COVID-19 affecting patients with obsessive–compulsive disorder? A longitudinal study on the initial phase of the pandemic in a Spanish cohort
Source: Eur Psychiatry. 2021 Jun 8;64(1):e45. doi: 10.1192/j.eurpsy.2021.2214 (PMC8280462; doi:10.1192/j.eurpsy.2021.2214)
Supplement: Supplementary file 1 [file S0924933821022148sup001.docx]

**Supplementary Table 1. Comparison of Covid-19 infection characteristics in the OCD and control cohorts**

| **Variables** | | **OCD**  **(n = 127)** | **Controls**  **(n = 237)** | **Χ^2^** | **p** |
| --- | --- | --- | --- | --- | --- |
| **Subject COVID-19 disease** | No | 119 | 214 | 1.1 | 0.5 |
|  | PCR confirmed | 2 | 4 |  |  |
|  | Clinical suspicion | 6 | 18 |  |  |
| **Subject hospital admission** | No | 125 | 234 | 0.4 | 0.5 |
|  | Yes | 2 | 2 |  |  |
| **Subject ICU admission** | No | 127 | 236 |  |  |
|  | Yes | 0 | 0 |  |  |
| **Family member Covid-19 Disease** | No | 104 | 170 | 4.4 | 0.1 |
|  | PCR confirmed | 15 | 40 |  |  |
|  | Clinical suspicion | 8 | 26 |  |  |
| **Family member Hospital admission** | No | 117 | 218 | 0.007 | 0.9 |
|  | Yes | 10 | 18 |  |  |
| **Family member ICU admission** | No | 123 | 227 | 0.1 | 0.7 |
|  | Yes | 4 | 9 |  |  |
| **Close friends Covid-19 disease** | No | 106 | 117 | 40.0 | ***<0.001*** |
|  | PCR confirmed | 21 | 119 |  |  |
| **Close friends hospital admission** | No | 117 | 187 | 9.9 | ***0.002*** |
|  | Yes | 10 | 49 |  |  |
| **Close friend ICU admission** | No | 123 | 204 | 10.0 | ***0.002*** |
|  | Yes | 4 | 32 |  |  |
| **Family or close friend death from Covid-19** | No | 121 | 217 | 1.4 | 0.2 |
|  | Yes | 6 | 19 |  |  |

*Abbreviations:* ICU, Intensive Care Unit; OCD, obsessive-compulsive disorder; PCR, polymerase chain reaction.
